# Unity or Diversity in Executive Functions: Examining the Three‐Factor Model in Young Children

**DOI:** 10.1002/pchj.70055

**Published:** 2025-09-27

**Authors:** Aleksander Veraksa, Morteza Charkhabi, Margarita Aslanova, Elena Dvorskaya, Vera Yakupova

**Affiliations:** ^1^ Department of Educational Psychology and Pedagogy Faculty of Psychology, Lomonosov Moscow State University Moscow Russia; ^2^ HSE University Moscow Russia; ^3^ Laboratory of Childhood Psychology and Digital Socialization, Federal Scientific Center for Psychological and Interdisciplinary Research Moscow Russia

**Keywords:** confirmatory factor analysis, executive functions, preschool age, school age, three‐factor model

## Abstract

Executive functions (EFs) as a set of cognitive processes play a crucial role in developing children's higher mental functions and academic success. Regardless of the number of studies conducted on EFs, current findings on the structure of cognitive functions as a whole or multifaceted construct are mixed. This study aims to evaluate and compare the latent factor structure of EFs in preschool‐aged children (5–7 years) and school‐aged children (7–9 years) to identify this structure across two age groups. The study involved 500 children divided into four age groups: senior kindergarten groups, preparatory kindergarten groups, first grade groups, and second grade groups. The participants were assessed using the NEPSY‐II neuropsychological battery and the Dimensional Change Card Sort task. The results revealed that a three‐factor model of EFs, comprising inhibitory control, working memory, and cognitive flexibility, best fits the data across all age groups. This suggests an earlier differentiation of EFs components, starting at the age of 5, which contradicts some previous studies proposing one‐ or two‐factor structures in preschool age. Correlation analysis showed statistically moderate relationships between EFs components, which weakened by the second grade, potentially indicating stabilization in EFs development during early school years. The findings support the unity and diversity model of EFs and emphasize the importance of conducting longitudinal research to clarify the factors influencing EFs development over time.

## 1 Introduction

Executive functions (EFs) as a set of general‐purpose control mechanisms modulate the operation of different cognitive processes and regulate the dynamics of human cognition (Miyake and Friedman 2012). EFs play a crucial role in the development of higher mental functions, such as attention, memory, and thinking, as emphasized by Vygotsky's perspectives (1982). According to Vygotsky (1982), the development of higher mental functions is closely related to the formation of behavioral control, which is a foundation for EFs. Recent research confirms that EFs not only contribute to the development of higher mental functions but also enable planning, control, and flexible adaptation to changing environmental conditions (Blair and Razza 2007; Diamond 2013). Specifically, the development of EFs is directly linked to success in solving tasks that require voluntary attention and logical thinking (Miyake and Friedman 2012; Best and Miller 2010). However, some authors emphasize that EFs are not a mechanism for the development of higher mental functions as they are a manifestation of them (Diamond 2013; Best and Miller 2010; Blair and Razza 2007). For instance, Diamond (2013) states that EFs are a set of cognitive processes that allow for the management of attention, behavior, and emotions. The development of EFs depends on more fundamental processes, such as the maturation of the prefrontal cortex and social interaction (Diamond 2013). Others argue that EFs are more of an indicator of general cognitive development rather than its mechanism (Best and Miller 2010). Earlier, Blair and Razza (2007) pointed out that EFs are the result of the interaction of biological and social factors rather than a primary mechanism of development.

EFs play a significant role in various aspects of life, including academic success and social competence over the years. Research indicates that the development of EFs during preschool age positively influences academic performance and social skills in school‐age children (Welsh et al. 2010; Best et al. 2011; Pascual et al. 2019; Veraksa et al. 2024). For example, children with more developed EFs demonstrate better abilities in focusing, attention, solving complex tasks, and adapting to new social situations, among others (Blair and Razza 2007; Diamond 2013).

An important theoretical and methodological issue in the study of EFs, as noted by Best and Miller (2010), is determining whether EFs represent a unified construct or consist of independent yet interrelated components. The answer to this question influences the approach to diagnosing and developing EFs. If EFs are a unified construct, their development can be viewed as a holistic process requiring general strategies. Conversely, if EFs consist of independent components, each of them necessitates separate diagnostic and developmental methods (Miyake and Friedman 2012; Diamond 2013). However, the study of EFs is not merely limited to this question. It is also crucial to understand how EFs interact with other cognitive processes and how their development affects overall mental functioning.

Despite studies that have attempted to identify the structure of EFs in children, reviewing current literature reveals that the structure of EFs in preschoolers and elementary school children requires further investigation. Some studies have suggested different structures at the same age. Therefore, the present study aims to fill this gap through examining and comparing various nested models. A direct comparison of different models of EF structure on a large sample allows us to determine the degree of differentiation of EF components based on age.

To date, various approaches to the structure of EFs have been presented in the scientific literature. In the three‐factor model proposed by Miyake et al. (2000), three components of EFs are identified: (1) inhibitory control, which ensures the regulation of attention, thinking, emotions, and behavior by suppressing dominant responses in favor of task requirements; (2) working memory (visual and auditory‐verbal), which allows for holding and mentally manipulating information; and (3) cognitive flexibility, which enables switching between tasks and considering them from different perspectives (Miyake and Friedman 2012; Diamond and Ling 2016). Alternative models have also been proposed, in which EFs are viewed as a unified construct reflecting a universal capacity for cognitive control (Wiebe et al. 2008; Friedman et al. 2008). Additionally, in the model proposed by Smith et al. (2004), two key components of EFs are highlighted: (1) inhibitory control and (2) cognitive flexibility. This diversity of approaches to the structure of EFs highlights the importance of studying their composition across different age groups, as age could be a potential a factor that may influence the dimensionality of the EFs components.

## 2 Development of EFs in Preschool and Elementary School

Each component of EFs has a unique developmental trajectory that may not coincide with the development of other components. For instance, research shows that inhibitory control, working memory, and cognitive flexibility develop during different age periods and at varying rates (Diamond 2013; Best and Miller 2010).

Inhibitory control refers to the ability to suppress prepotent responses, when necessary, to resist interference from distracting stimuli, and to inhibit inappropriate or unwanted behaviors (Best and Miller 2010). The assessment of inhibitory control begins at the age of 3. As shown in reviews dedicated to the development and diagnostic methods of inhibitory control (Simpson and Riggs 2005; Huizinga et al. 2006), it is at this age that children start to successfully perform tasks such as the “Child Stroop Test”, the “Inhibition” subtest of the NEPSY II neuropsychological battery, and the “Day‐Night task” (Wiebe and Karbach 2018; Best and Miller 2010; Korkman et al. 2007). These methods involve tasks where the child must suppress an automatic response (e.g., naming pictures in reverse or ignoring distracting stimuli) in favor of following a given rule. Inhibitory control develops and improves between the ages of 5 and 8, as evidenced by a reduction in errors and an increase in the speed of completing tasks requiring cognitive and behavioral control (Romine and Reynolds 2005; Huizinga et al. 2006; Zakharova and Machinskaya 2023). Inhibitory control reaches maturity during adolescence and early adulthood, reflected in the ability to effectively solve complex tasks requiring sustained focus and suppression of distractions (Huizinga et al. 2006; Diamond 2013).

Working memory refers to the ability to maintain and manipulate information over short periods of time, which is essential for guiding behavior and decision‐making in the face of competing demands (Luna 2009). The assessment of working memory becomes feasible starting at the age of 3–4 years through tasks involving voluntary recall, which are based on presenting the child with a sequence of stimuli (e.g., numbers, words, or images) that they must remember and reproduce in the correct order, similar to the “SPAN” test (Conway et al. 2005). Additionally, subtests such as the “Memory for Design” and “Sentence Repetition” from the NEPSY II neuropsychological battery (Korkman et al. 2007) are widely used for diagnosing working memory. In these tasks, children are required to memorize and reproduce images or sentences with gradually increasing complexity (Gathercole et al. 2004; Alloway and Alloway 2010). Using sequence recall tasks, it has been demonstrated that by the age of 4, children can retain two to three elements simultaneously in their memory (Gathercole et al. 2004). By the age of 6, the capacity of working memory increases to four to five elements (Simmering and Perone 2013). However, visual and auditory‐verbal memory develop at slightly different rates: auditory‐verbal memory typically allows for retaining more elements than visual memory, which is associated with differences in information encoding (Gathercole et al. 2004; Simmering and Perone 2013).

Cognitive flexibility refers to the ability to shift between mental sets, tasks, or strategies in response to changing environmental demands (Zelazo et al. 2003; Zelazo 2006). The assessment of cognitive flexibility in children begins at the age of 4. Diagnostic methods for cognitive flexibility typically involve tasks where the child is presented with stimuli that must be sorted according to one criterion (e.g., color) and then another (e.g., shape), requiring the child to switch between rules. The most commonly used method for assessing cognitive flexibility is the “Dimensional Change Card Sort” (DCCS) task (Zelazo 2006; Davidson et al. 2006). Qualitative improvements in cognitive flexibility, such as a reduction in errors and an increase in task completion speed, are observed during the preschool years (ages 5–7), when children begin to successfully solve more complex tasks requiring the simultaneous consideration of multiple rules (Zelazo et al. 2013; Moriguchi 2018). For example, in the third trial of the “Inhibition” subtest (NEPSY II), the child is asked to perform a task requiring switching between two rules: they must name white images in reverse (e.g., calling circles squares and squares circles) while naming black images normally (Korkman et al. 2007). Another method for assessing cognitive flexibility is the “Trail Making Test” (child version), where the child must connect numbers and letters in a specific sequence (e.g., 1‐A; 2‐B; 3‐C, etc.) (Reitan 1958). The “Attention Switching Task” requires the child to respond to changing stimuli (e.g., color or shape) according to a predefined rule, demanding constant attention switching and adaptation to changing conditions (Davidson et al. 2006).

The development of all three components of EFs is interrelated. Working memory and inhibitory control exhibit a mutually supportive relationship: the development of one component facilitates the development of the other (Diamond 2013). Their joint progress contributes to the formation of cognitive flexibility, particularly, evident in senior preschool‐aged and elementary school‐aged children (Diamond 2013; Moriguchi 2018). Despite the connections between the components of EFs, their development remains a complex and independent process. The varying developmental trajectories of EFs support A. Miyake's model, which posits the relative differentiation of the three components of EFs, although the degree of unity and diversity may change across different ages (Garon et al. 2008; Best and Miller 2010; Chichinina and Gavrilova 2022; Almazova et al. 2024).

The critical age for the development of EFs is the senior preschool age (ages 5–7), during which a significant leap in cognitive development occurs (Best and Miller 2010; Diamond 2013). According to Elkonin's theory, each age period is characterized by a unique social situation of development, determined by the circle of significant others and the nature of interactions with them (Elkonin 1971). This situation defines the leading activity of the child, within which key developmental achievements of the age are formed. By the end of the stage, these achievements lead to a crisis: the child changes, but the social situation remains the same, creating a contradiction that stimulates the transition to a new developmental stage (Elkonin 1971; Vygotsky 1984).

In senior preschool age, the leading activity is role‐playing (Elkonin 1978), which promotes the development of symbolic thinking, imagination, and theory of mind (Piaget 1954; Veraksa 2011). By the age of 7, children develop a new interest in exploring the objective world, associated with the transition to learning activity (Elkonin 1971). This transition is accompanied by a significant leap in the development of EFs. Research shows that at this age, children begin to demonstrate higher levels of inhibitory control (Best and Miller 2010; Moriguchi 2018), the ability to retain and use information in working memory (Gathercole et al. 2004; Luna 2009), and improved cognitive flexibility (Zelazo et al. 2013), necessary for adapting to changing demands. The transition to school represents an important social situation, as the demands on EFs increase significantly. This developmental leap is associated not only with cognitive changes but also with changes in the social situation of development.

Furthermore, some studies indicate that the type of representation (verbal and nonverbal) plays a key role in the development of EFs (Veraksa 2011; Grobe et al. 2024). In preschool age, nonverbal representation dominates, which is associated with the immaturity of language skills. Children perform better on tasks requiring visuospatial analysis and inhibitory control (Zelazo 2006; Simmering and Perone 2013). With the transition to school, verbal representation begins to play a more significant role, as academic tasks often require verbal encoding. This shift in representation type contributes to the development of working memory and cognitive flexibility. For instance, studies demonstrate improved performance on verbal tasks in elementary school age (Gathercole et al. 2004; Almazova et al. 2018; Joukova et al. 2023).

Thus, the development of EFs in senior preschool age is not only a result of cognitive maturation but also a consequence of changes in the social situation of development and the child's leading activity. In turn, changes in the social situation of development stimulate a redistribution of the roles of verbal and nonverbal representations, influencing the further development of EFs.

## 3 The Factor Structure of EFs in Childhood

The three‐factor structure of EFs advocated by Miyake et al. (2000) has now become the principal paradigm for grasping EFs. It amalgamates theoretical views on neurocognitive processes with empirical data on the consolidation and variety of EFs. From a theoretical perspective, this framework corresponds with ideas of functional differentiation in the prefrontal cortex (Roberts et al. 1994; Welsh et al. 1991), where various cortical zones are responsible for distinct facets of behavioral control. Neuropsychological research on individuals with frontal lobe damage reinforces this distinction by showing separations among EFs facets (Casey et al. 1997). Empirically, the three‐factor structure has been corroborated through investigation with adults and older children, affirming that the three‐factor structure (inhibitory control, working memory, and cognitive flexibility) aligns most accurately with the data. The findings show moderate correlations (*r* = 0.42–0.63), suggesting they have some relationship but also act independently (“Unity and Diversity”) (Miyake et al. 2000). The study utilizing structural equation modeling (SEM) has determined that each individual component of EFs contributes distinctively to the execution of complex cognitive tasks. This discovery holds significant practical relevance for comprehending cognitive mechanisms and aids in tackling the task contamination issue—the muddled impact of various cognitive activities on test outcomes (Miyake et al. 2000). However, the structure appears less differentiated in younger preschool‐aged children. Studies (Fuhs and Day 2011; Wiebe et al. 2008) indicate that EFs may present as a unitary construct in early growth, probably due to the immaturity of the prefrontal cortex and the progressive specialization of its parts as children age (Garon et al. 2008).

Indeed, the view that during preschool age, the components of EFs—inhibitory control, working memory, and cognitive flexibility—are not sufficiently differentiated is widely believed. This is reflected in the predominance of a one‐factor structure of EFs in various studies (Wiebe et al. 2008; Hughes et al. 2010; Shing et al. 2010; Willoughby et al. 2010; Fuhs and Day 2011). However, some research also supports two‐factor models, where inhibitory control is identified as a separate factor, while working memory and cognitive flexibility are combined into another one (Usai et al. 2014; Monette et al. 2015; Michel and Bimmüller 2023). Less common are two‐factor models where working memory is isolated as a separate factor, and inhibitory control and cognitive flexibility are combined (Lee et al. 2013; van der Ven et al. 2013). Working memory, inhibitory control, and cognitive flexibility are exposed as three independent factors in preschool age in few studies. Thus, studies indicate that a one‐factor structure of EFs is most frequently observed in preschool age. It can be explained by the immaturity of cognitive processes and their close interrelation during early developmental stages. Another possible explanation is based on the fact that at this age, the social situation of a child's development does not impose relevant demands on the development of EFs.

As children transit to school, the structure of EFs begins to differentiate, which is reflected in the increased prevalence of two‐ and three‐factor models (Fuhs and Day 2011) in research. For example, two‐factor structures have been identified, including inhibitory control and a combined factor of working memory and cognitive flexibility (Huizinga et al. 2006; Lee et al. 2013; van der Ven et al. 2013). At the same time, some studies still report a one‐factor structure (Xu et al. 2013; Brydges et al. 2014; Johann et al. 2022).

Differences in the factor structure of EFs may be attributed to several reasons. For instance, the use of different methodologies across studies makes it challenging to compare results (Bardikoff and Sabbagh 2017). Additionally, some studies do not assess all EF components (Wiebe et al. 2008; Shing et al. 2010). Moreover, some research does not present two‐ or three‐factor models that might demonstrate better fit indices (Hughes et al. 2010).

In the review section of the article, studies were examined that demonstrate heterogeneity in the composition and relationship of EF constructs even within the same age group. Considering the described features and limitations of previous research, the current study aims to evaluate the factor structure of EFs in samples of senior preschool‐aged (5–6 and 6–7 years) and elementary school‐aged children (7–8 and 8–9 years). The assessment of EF components was conducted using subtests from the “NEPSY II neuropsychological battery” (Korkman et al. 2007) and the “DCCS” task (Zelazo 2006), which are widely used in international studies, successfully tested on Russian samples (Veraksa et al. 2020), and allow for the evaluation of all EF components: inhibitory control, working memory, and cognitive flexibility. The selected age range is critical for EF development and is characterized by the transition from kindergarten to school, which requires a significant increase in the level of executive functioning in children (Diamond 2013). Building on Miyake et al. (2000), we formulated the two following research hypotheses to clarify on the dimensionality of the EF components across preschool and school‐aged children:

Hypothesis 1 *A one‐factor model of EFs will statistically represent stronger fit indices in senior preschool age compared to other models*.

Hypothesis 2 *A three‐factor model of EFs will statistically represent stronger fit indices in elementary school‐aged children compared to other models*.

## 4 Method

### 4.1 Procedure

The study involved 500 Russian children. Among them, 238 were preschoolers: 118 children from senior kindergarten groups (mean age = 70.6 months, SD = 4.5, range = 61–79 months, 50% boys) and 120 children from preparatory groups (mean age = 82 months, SD = 4.75, range = 65–89 months, 53.3% boys). The remaining 262 participants were first‐ and second‐grade elementary school children: 151 first‐graders (mean age = 95.7 months, SD = 3.85, range = 86–103 months, 49% boys) and 111 second‐graders (mean age = 102 months, SD = 4.03, range = 89–112 months, 47.7% boys).

In the Russian Federation, children start school between the ages of 6.5 and 8 years, which aligns with international standards. In senior kindergarten groups, which usually start from 4.5 to 5 years old and end at 6.5 to 7 years old, education in kindergartens plays an important role in preparing children for school by developing cognitive, social, and emotional skills. We intentionally included children attending kindergarten in the sample because research indicates that children who do not attend kindergarten may face challenges in adapting to school requirements and developing EFs (Diamond 2013; Pascual et al. 2019). Thus, including preschoolers in the sample allows for a more accurate assessment of the factor structure of EFs during the critical transition period from kindergarten to school.

### 4.2 Participants

The assessment of EFs development in children was conducted in the spring of 2024 among senior and preparatory kindergarten groups of state preschool educational institutions and first‐ and second‐grade classes of state general education schools in Moscow. All tasks were administered individually in a quiet, separate room within the kindergarten or school. Senior preschoolers (senior and preparatory kindergarten groups) participated in two diagnostic sessions lasting 15–25 min each, while elementary school children (first‐graders and second‐graders) completed one session lasting 30–45 min. This is due to the state educational standards. Preschoolers cannot practice for more than 20 min to avoid cognitive overwork. Elementary school children can practice for 40–45 min, which corresponds to the lesson time. The assessments were carried out by trained and certified research team members. Participation in the study was subject to filling and signing a written parental consent form. Children's data were anonymized to ensure the confidentiality of data. The study was also approved by the Ethics Committee of the Faculty of Psychology at Lomonosov Moscow State University (approval number: 2023/18). Written informed consent was obtained from the legal guardians of all participants.

### 4.3 Measures

To assess the development of EF components (inhibitory control, working memory, and cognitive flexibility) in senior preschoolers (senior and preparatory kindergarten groups) and elementary school children (first‐graders and second‐graders), a battery of subtests from the NEPSY‐II neuropsychological diagnostic assessment was used. This assessment has been validated and used on Russian samples (Korkman et al. 2007; Veraksa et al. 2020).

#### 4.3.1 Inhibitory Control

The NEPSY‐II subtest of the “Inhibition” was used to measure inhibitory control. It includes two series of white and black figures: geometric shapes (circle, square) and arrows pointing in different directions (up, down). Children completed two tasks: a naming task (naming the figures as quickly as possible) and an inhibition task (naming the figures inversely, e.g., saying “square” when a circle is shown). We used time (more time—lower score) and composite score as important measures of the effectiveness of the methodology. Composite scores for inhibitory control were calculated based on the child's exact age in months, the number of errors (corrected and uncorrected), and task completion time. The maximum score for this subtest is 19.

#### 4.3.2 Visual Working Memory

The NEPSY‐II subtest of “Memory for Designs” was used to measure visual working memory. Children were shown cards with images arranged in a 4 × 4 grid. After 10 s, the cards were removed, and the child had to select and place only the previously shown cards in the correct grid cells among distractors and previously seen cards. The subtest consists of four trials, with the number of cards increasing from four to eight per grid. The final score for visual working memory was calculated based on the sum of points for content (correct card selection), location (correct placement in the grid), and bonus points (correct card selection and placement). The maximum score for this subtest is 120.

#### 4.3.3 Auditory‐Verbal Working Memory

The NEPSY‐II subtest of “Sentence Repetition” was administered to assess verbal working memory. It consists of 17 sentences, with complexity increasing progressively. The examiner read each sentence in a neutral tone and at a calm pace, and the child repeated it. Correct repetitions earned 2 points, while errors (e.g., adding, omitting, or substituting words) resulted in a deduction of 1 point. The total score for auditory‐verbal working memory was the sum of points across all sentences, with a maximum score of 34.

#### 4.3.4 Cognitive Flexibility in Preschool Children

The DCCS task (Zelazo 2006) was used to assess cognitive flexibility. Children were asked to sort cards according to three rules: by color, by shape, and by switching between these rules (sorting by color if the card had a frame and by shape if it did not). The total score for cognitive flexibility was the sum of points across all three trials (maximum: 6, 6, and 12 points for each trial), with 1 point awarded for each correctly sorted card. The maximum score for this task is 24.

#### 4.3.5 Cognitive Flexibility in Elementary School Children

The third task of the “Inhibition” subtest of NEPSY‐II was used to evaluate cognitive flexibility. The change in method is because, according to the DCCS procedure, it is only used for up to 7 years' children. In the cognitive flexibility task, children were asked to name shapes (circles and squares) of a specific color inversely (naming black shapes normally and white shapes inversely). The cognitive flexibility task was administered after the inhibitory control tasks with shapes and arrows. We used time (more time—lower score) and composite score as important measures of the effectiveness of the methodology. Composite scores for cognitive flexibility in elementary school children were calculated similarly, considering the child's exact age in months, the number of errors, and task completion time. The maximum score for this subtest is 19.

### 4.4 Data Analysis

Data analysis was performed using Microsoft Excel 2010 and Jamovi 2.3.21.0. The exploratory module was used to calculate descriptive statistics (such as means and standard deviations). Since the distribution of most variables differs significantly from normal (confirmed by the results of the Shapiro–Wilk and Kolmogorov–Smirnov tests), and unequal variances were found in a number of the compared subgroups, nonparametric methods or corrections to parametric methods were used for statistical analysis. The *t* test module for independent samples and ANOVA with a nonparametric Welch correction were used for pairwise comparison of the average values of various EFs in age groups. The regression module allowed us to evaluate correlations between different regulatory functions (Spearman's criterion). Confirmatory factor analysis (CFA) was used to test structural models. Visualization of structural models was carried out in the online service draw.io.

## 5 Results

### 5.1 Descriptive Statistics

Table 1 presents the descriptive statistics for variables measuring EFs in senior preschool (senior and preparatory kindergarten groups) and elementary school children (first‐graders and second‐graders) across age groups. The results of EFs assessment in children align with age norms previously found in studies that had investigated senior preschoolers and elementary school children (e.g., Almazova et al. 2024). In general, the descriptive statistics reflect a trend toward an increase in EFs from preschool age to the beginning of the first grade, with the most crucial improvement observed in visual working memory. In the second grade, EFs are already growing less rapidly.

**TABLE 1:** Descriptive Statistics of Executive Functions Across Preschool and Elementary School Children.

Executive Functions	Preschool (*n* = 238)				Elementary School (*n* = 262)			
	SKG (*n* = 118)		PG (*n* = 120)		FG (*n* = 151)		SG (*n* = 111)	
	*M*	SD	*M*	SD	*M*	SD	*M*	SD
Visual Working Memory	72.4	20.9	82.8	21.7	104.9	23.8	103.1	24.6
Auditory‐Verbal Working Memory	18.8	3.61	19.5	4.64	21.6	4.29	21.5	4.2
Inhibitory Control (Time)	122	31.8	106.1	23.4	91.5	19.3	87.5	17.5
Inhibitory Control (Composite Score)	11.4	3.17	10.8	3.12	11.3	2.84	11.3	2.9
Cognitive Flexibility	20.2	2.73	21.2	2.51	129	29.4	124	22.4
Cognitive Flexibility (Composite Score)	—	—	—	—	11	2.96	10.7	3.23

Abbreviaitons: FG = First Grade Group; PG = Preparatory Group; SG = Second Grade Group; SKG = Senior Kindergarten Group.

Table 2 presents the results of the analysis of differences between groups of children (senior kindergarten group, preparatory group, first‐graders, second‐graders) in terms of EFs. Nonparametric tests including Mann–Whitney *U* and ANOVA with nonparametric Welch's correction test were used to test the differences in EFs across children's groups. The post hoc Games‐Howell test was used to assess pairwise comparisons, allowing for unequal variance (as independent of equality of variance). The Mann–Whitney *U* test was used to compare cognitive flexibility in pairs in preschool and school‐age subgroups due to the use of different techniques (see Section 4.3). The results suggest significant changes in EFs levels over time. However, no significant differences were found between the first and second graders for any of the EFs.

**TABLE 2:** Results of pairwise comparisons of executive functions across age groups.

Variables	*t*	Pairwise comparisons**
Visual working memory	61.98***(Welch)	SKG < PG SKG < FG SKG < SG PG < FG PG < SG
Auditory‐verbal working memory	15.82***(Welch)	SKG < PG SKG < FG SKG < SG PG < FG PG < SG
Inhibitory control (time)	45.10***(Welch)	SKG < PG SKG < FG SKG < SG PG < FG PG < SG
Cognitive flexibility	5490**(U)	SKG < PG

Abbreviations: FG = first‐grade group; PG = preparatory group; SG = second‐grade group; SKG = senior kindergarten group.

** *p* < 0.01.

*** *p* < 0.001.

### 5.2 Correlation Analysis

A correlation analysis was conducted to draw correlation matrices among research variables. Table 3 tabulates the correlation coefficients. Only significant correlations are presented in the table. As the table shows, positive and negative correlations were found between variables measuring EFs at each age, most of which can be considered stable.

**TABLE 3:** Correlations among executive functions across age groups.

Variables pair	SKG	PG	FG	SG
Visual WM and auditory‐verbal WM	0.30***	0.41***	0.33***	—
Visual WM and inhibitory control (time)	−0.28**	−0.48***	−0.24**	−0.23**
Visual WM and inhibitory control (composite)	0.26**	0.40***	0.25**	0.29**
Visual WM and cognitive flexibility (preschool)	0.20*	0.36***	—	—
Visual WM and cognitive flexibility (school time)	—	—	0.62***	0.66***
Visual WM and cognitive flexibility (school composite)	—	—	0.20	0.43***
Auditory‐verbal WM and inhibitory control (time)	—	−0.23**	−0.21**	—
Auditory‐verbal WM and inhibitory control (composite)	0.22*	0.24**	0.20**	—
Auditory‐verbal WM and cognitive flexibility (preschool)	0.40***	0.40***	—	—
Inhibitory control (time) and cognitive flexibility (preschool)	0.20*	0.23**	—	—
Inhibitory control (time) and cognitive flexibility (school time)	—	—	−0.23	−0.31
Inhibitory control (time) and cognitive flexibility (school composite)	—	—	−0.35**	−0.39**

Abbreviations: FG = first‐grade group; PG = preparatory group; SG = second‐grade group; SKG = senior kindergarten group; WM = working memory.

* *p* ≤ 0.05.

** *p* ≤ 0.01.

*** *p* ≤ 0.001.

According to the results, visual working memory is positively associated with auditory‐verbal working memory in age groups up to the second grade (*r* [0.302–0.406]), as well as with inhibitory control (*r* [−0.275–0.403]) and cognitive flexibility (*r* [0.197–0.359]) across all age groups. The correlation strength is higher in the preparatory groups.

Auditory‐verbal working memory is positively associated with inhibitory control in children up to the second grade (*r* [0.204–0.236]) and with cognitive flexibility in all age groups (*r* [−0.283 to 0.433]) except first‐graders. Inhibitory control (*r* [−0.226 to 0.236]) is associated with cognitive flexibility in all age groups. The correlation strength is higher in the preparatory groups and second grade. Thus, the relationships between EF components are consistent across preschool and elementary school ages. The exception is second‐graders, for whom the correlation between visual and auditory‐verbal working memory, as well as between auditory‐verbal working memory and inhibitory control, disappears. Additionally, the correlation between auditory‐verbal working memory and cognitive flexibility disappears in first‐graders.

### 5.3 Factor Structure of EFs

To assess the factor structure of EFs, a CFA was conducted. The results of the CFA are tabulated in Table 4. Models were tested separately for each age group. One‐, two‐, and three‐factor structures of EFs were developed and compared. The three‐factor models were found to have the best fit for all age groups, and they were the most parsimonious models. For each age group, the chi‐square test values for the one‐ and two‐factor models did not indicate a good fit to the data, unlike the three‐factor models.

**TABLE 4:** Goodness of fit indices for executive functions models across age groups.

Age group	Model	*χ* ^*2*^	df	*p*	CFI	RMSEA	BIC
SKG (*n* = 118)	One‐factor	80.8	9	< 0.001	0.797	0.260	4357
	Two‐factor	278	8	< 0.001	0.237	0.535	4560
	Three‐factor	9.48	6	0.148	0.991	0.07	4289
PG (*n* = 120)	One‐factor	80.6	9	< 0.001	0.852	0.258	4261
	Two‐factor	66.0	8	< 0.001	0.880	0.246	4251
	Three‐factor	8.16	5	0.148	0.993	0.07	4208
FG (*n* = 151)	One‐factor	76.3	9	< 0.001	0.701	0.223	6442
	Two‐factor	64.9	8	< 0.001	0.748	0.217	6435
	Three‐factor	7.8	4	0.099	0.983	0.079	6398
SG (*n* = 111)	One‐factor	44.6	9	< 0.001	0.800	0.189	4663
	Two‐factor	44.4	8	< 0.001	0.795	0.203	4668
	Three‐factor	4.18	4	0.382	0.999	0.02	4646

*Note*: Models with the best fit indices are highlighted in bold. Acceptable fit thresholds for CFA indices were CFI ≥ 0.95, RMSEA ≤ 0.05, *p*‐value *χ* ^2^ ≤ 0.05, and lower BIC values indicate better fit.

Abbreviations: BIC = Bayesian Information Criterion; CFI = comparative fit index; df = degrees of freedom; FG = first‐grade group; *p* = significance; PG = preparatory group; RMSEA = root mean square error of approximation; SG = second‐grade group; SKG = senior kindergarten group; *χ* ^2^ = Chi‐square.

All standardized factor loadings, their standard errors, and standardized residual deviations for each indicator and age group are presented in Table 5 (as reported by Jamovi output). The residual deviations exceed one, which is a common feature of the Jamovi Program normalization procedure. The obtained values confirm the relationship of each indicator with its corresponding latent factor.

**TABLE 5:** Standardized factor loadings, standard errors, and residual variances for executive functions across age groups.

Variable	Factor	Factor loading (SKG)	SE (SKG)	RV (SKG)	Factor loading (PG)	SE (PG)	RV (PG)	Factor loading (FG)	SE (FG)	RV (FG)	Factor loading (SG)	SE (SG)	RV (SG)
Visual working memory	Working memory	0.457	2.360	0.543	0.781	2.397	0.219	0.721	5.193	0.279	0.307	3.275	0.693
Auditory‐verbal working memory		0.666	0.498	0.334	0.544	0.477	0.456	0.403	0.695	0.597	0.497	0.720	0.503
Inhibitory control (time)	Inhibitory control	0.698	4.422	0.302	0.770	2.378	0.23	0.997	8.923	0.003	0.687	2.115	0.313
Inhibitory control (composite score)		−0.889	0.486	0.111	−0.685	0.313	0.315	−0.356	0.343	0.644	−0.718	0.306	0.282
Cognitive flexibility	Cognitive flexibility	0.915	0.200	0.085	0.964	0.166	0.036	0.953	7.159	0.047	0.657	2.422	0.343
Cognitive flexibility (composite score)		1.039	0.199	—	1.012	0.169	—	−0.445	0.299	0.555	−0.860	0.329	0.14

Abbreviations: Factor loading = standardized factor loading; FG = first grade group; PG = preparatory group; RV = standardized residual variance (as reported by Jamovi output); SE = standard error; SG = second‐grade group; SKG = senior kindergarten group.

**FIGURE 1:**
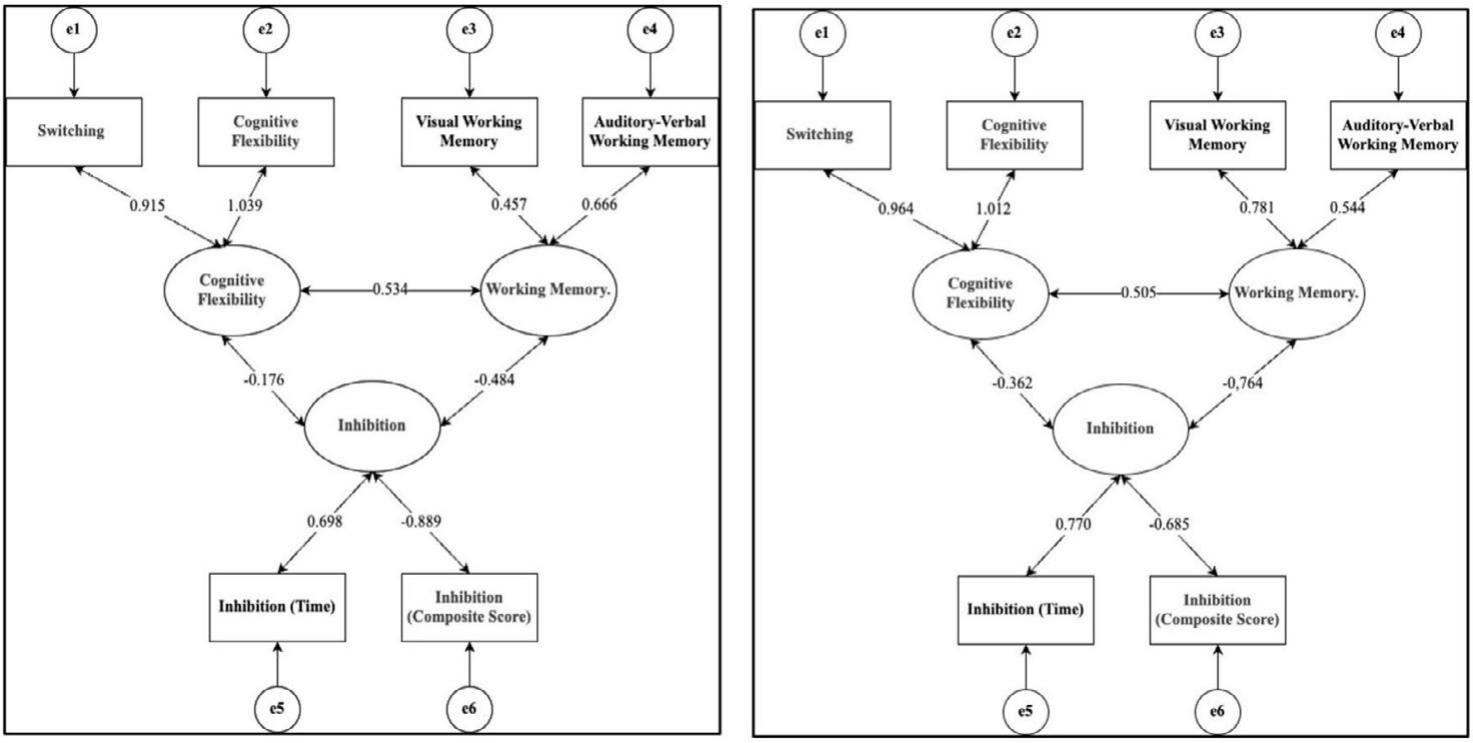
Latent factor structure of executive functions in senior preschool age. *Note*: Left = senior kindergarten group, right = preparatory group. Negative correlations are associated with the reverse scoring of certain scales.

**FIGURE 2:**
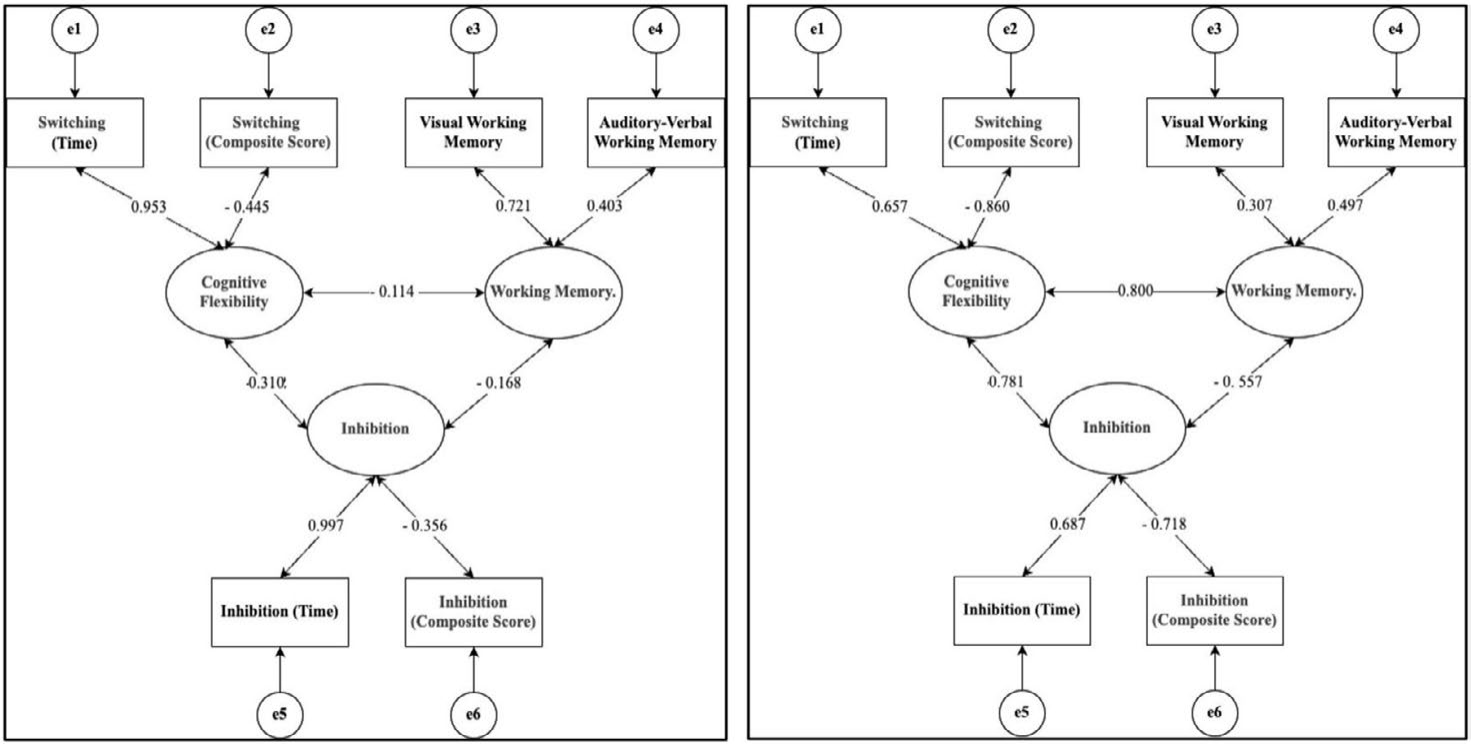
Latent factor structure of executive functions in elementary school age. *Note*: Left = first grade group, right = second grade group. Negative correlations are associated with the reverse scoring of certain scales.

Figures 1 and 2 illustrate a graphical demonstration of the relations between the components EFs and their factor loadings. The calculated fit indices for the three‐factor models demonstrate acceptable values after accounting for residual correlations between items. In the latent factor structure of EFs for senior preschool age (senior and preparatory kindergarten groups), the variance for the cognitive flexibility factor exceeds 100%.

## 6 Discussion

This study inspected the changes of EFs across children age groups to clarify further on the dimensionality of these functions as a uni‐ or multifactor construct. This study focused on assessing EFs in senior preschool and school‐aged children.

### 6.1 Three‐Factor EFs Model in Children

A key finding of this study provided empirical evidence for the three‐factor structure of EFs that was supported by comparing the fit indices of three nested models (Table 4). The three‐factor model was found to be the most parsimonious model among the tested models for both senior preschool‐aged and elementary school‐aged children. This finding is important, suggesting an earlier differentiation of all three EFs components starting from the age of 5. This finding aligns with international studies that have identified two‐factor models of EFs as the beginning of their differentiation in senior preschool and elementary school children (Miller et al. 2012; Lee et al. 2013; van der Ven et al. 2013; Usai et al. 2014; Monette et al. 2015; Simanowski and Krajewski 2019; Michel and Bimmüller 2023; Huizinga et al. 2006), as well as three‐factor models (Lehto et al. 2003; Arán‐Filippetti 2013; Duan et al. 2010; Rose et al. 2012). Following the study of Lehto et al. (2003), who identified a three‐factor structure of EFs in children aged 8–13 years across a combined age range, our study revealed a three‐factor structure of EFs in each age group from 5 to 9 years.

Despite the fact that many studies report a one‐ or two‐factor structure of EFs in preschool children, our results suggested a three‐factor structure as early as the age of 5. This finding, in addition, may explain: (1) differences in measurements (we used the NEPSY‐II and DCCS tests) and (2) sampling approach and cultural context. Children are exposed to structured educational activities from an early age. This may contribute to the earlier functional specialization of EFs due to environmental requirements.

Our findings may support theories that environmental factors such as education and gaming can influence the timing of functional differentiation. According to the “unity and diversity” model of Miyake and Friedman (2012), the degree of independence of EFs components will gradually increase with age. The presented results expand on previous theoretical models, emphasizing the role of context, methodology, and early experience. This brings new data to the discussion about the trajectory of EFs and highlights the importance of cross‐cultural and longitudinal research.

### 6.2 Contradictory Evidence and Methodological Factors

The results of this study diverge from data indicating a one‐factor structure of EFs in senior preschool‐aged and elementary school‐aged children (Wiebe et al. 2008, 2011; Hughes et al. 2010; Shing et al. 2010; Willoughby et al. 2010, 2012; Fuhs and Day 2011; Xu et al. 2013; Brydges et al. 2014; Johann et al. 2022; Laureys et al. 2022; Michel and Bimmüller 2023; Grobe et al. 2024). The three‐factor structure of EFs identified in children aged 5–9 years in this study may be related to the methodological approach used, including the absence of model fitting and outlier exclusion, which distinguishes our work from some other studies (Huizinga et al. 2006; Lee et al. 2013; van der Ven et al. 2013; Brydges et al. 2014; Laureys et al. 2022). It was shown that an unrestricted model demonstrates better fit indices than a restricted one (Brydges et al. 2014).

The identification of the three‐factor structure of EFs in children at age 5 can become the basis for new educational practices and the development of research methods. Kindergarten educational programs can target the development of individuals' EFs components during regular classes. In addition, it is possible to develop more detailed diagnostic tools to identify children with underdeveloped EFs at earlier ages. This would make it possible to identify more precisely the areas in which the child will need support. Then the intervention will be directed not only at a specific component of the EFs, but even on its specific cognitive side. Taking into account the three‐factor nature of EFs will enable educators and researchers to create effective tools and interventions to improve children's learning outcomes and their social abilities.

### 6.3 Neuroanatomical Support

The revealed three‐factor structure of EFs finds convincing confirmation in the ideas about the functional organization of the prefrontal cortex. For example, the dorsolateral prefrontal cortex is associated with working memory, the ventrolateral cortex with inhibitory control, and the anterior cingulate cortex with cognitive flexibility (Roberts et al. 1994; Welsh et al. 1991). In addition, selectivity of cognitive impairment has been shown in patients with localized brain lesions, and this is able to confirm the neuroanatomical independence of EFs (Casey et al. 1997). This agrees well with the structure of EFs that we have identified because it emphasizes the presence of an anatomical basis for the formation and functioning of EFs.

### 6.4 Task Impurity and Measurement Constraints

One of the challenges in studying the factor structure of EFs in childhood is the low construct validity of tasks used to assess EFs (Brocki and Bohlin 2004; Best and Miller 2010; Bardikoff and Sabbagh 2017; Karr et al. 2018). To date, there are no tasks that assess each EFs component in isolation—tasks often require non‐EFs cognitive processes as well (Best and Miller 2010; Bardikoff and Sabbagh 2017). For example, tasks may involve visual or auditory information processing and require verbal or motor responses (van der Ven et al. 2013). This phenomenon is known as the “task impurity effect,” which refers not only to the involvement of non‐EFs cognitive processes in task performance but also to the fact that EFs processes rarely operate in isolation (Miyake et al. 2000; Huizinga et al. 2006). To address this issue, Miyake et al. (2000) proposed using multiple tasks to measure each EFs component and applying a latent variable approach to extract variance common to these tasks.

In this study, a single task was used to assess cognitive flexibility and inhibitory control. It has been argued that using a single task reduces the effectiveness of latent variable analysis, as the variance structure may appear more homogeneous than it actually is (Hughes et al. 2010; Wu et al. 2011; McAuley and White 2011; Lee et al. 2013). However, some studies have shown that multiple tasks aimed at assessing inhibitory control may not form a single factor (Huizinga et al. 2006; van der Sluis et al. 2007). Thus, the risk remains that the factor structure of EFs demonstrated in any study may depend on the specific tasks used, even when multiple tasks are employed.

In the study by Michel and Bimmüller (2023), the sensitivity of models to the choice of tasks was demonstrated. By excluding indicators from certain tests with high correlations, they obtained both one‐ and two‐factor models on the same sample. It is possible that the three‐factor structure identified in this study emerged because simpler tasks were used, requiring greater involvement of the EFs components being assessed. Complex tasks are more likely to exhibit the task impurity effect (Huizinga et al. 2006; Lee et al. 2013). For example, the “Wisconsin Card Sorting Test” (WCST) simultaneously engages multiple EFs components: inhibitory control and cognitive flexibility (Huizinga et al. 2006; Garon et al. 2008; Best et al. 2011). However, in the study by Huizinga et al. (2006), WCST performance was predicted by working memory capacity, possibly due to the task's procedural characteristics, as the rules were not repeated with each card presentation. In our study, the “DCCS” task (Zelazo 2006) repeated the rules with each presentation, suggesting that the results of EFs factor structure studies may be sensitive to the instructions given for each specific task (Stuss and Alexander 2000). In this study, cognitive flexibility and inhibitory control in elementary school children were assessed using different tasks within the same subtest. It has been suggested that the shared context of these tasks may obscure differences between the two EFs components, reducing the cost of cognitive flexibility and affecting the overall variance (Lee et al. 2013). Referring to the study by Lee et al. (2013), which used both separate subtests and tasks with individual trials assessing these two EFs components—the “Flanker task” and the “Simon task”—it was found that cognitive flexibility did not emerge as a separate factor, regardless of whether it was assessed in a separate subtest or within a block of inhibitory control tasks, across age groups from 5 to 13 years.

The analysis of EFs structure also depends on the scoring system (van der Ven et al. 2013). Assessing cognitive flexibility and inhibitory control requires considering both accuracy and speed (van der Ven et al. 2013). Some studies of EFs factor structure in children do not use speed indicators (Willoughby et al. 2010; Fuhs and Day 2011). In this study, a three‐factor structure of EFs was identified, incorporating both accuracy and speed measures.

Additional reasons why a three‐factor structure of EFs may not have been identified in other studies include the presentation of stimuli in the same format across tasks, such as always presenting pairs of stimuli and requiring verbal responses (van der Sluis et al. 2007). The format of the diagnostic procedure may also play a role. In some studies, school children performed tasks in small groups of two to three participants (Huizinga et al. 2006; Lee et al. 2013; Brydges et al. 2014). In our study, tasks were administered individually to both senior preschool (senior and preparatory kindergarten groups) and elementary school children.

### 6.5 Developmental Trajectory and Age‐Related Changes

Thus, the three‐factor model was found to be the best fit for the studied age groups. The latent factor structures are represented by correlated EFs components: inhibitory control, working memory, and cognitive flexibility. The presence of correlations is shown not only for the factors (Figures 1 and 2), but also for the results of the techniques measuring components of EFs (Table 3). This is an important observation because correlations between factors depend on their weights in the aggregation, whereas components do not contain this constraint. This result supports the three‐factor unity and diversity model of EFs, not only in adults (Friedman and Miyake 2004; Miyake et al. 2000) but also in senior preschool‐aged and elementary school‐aged children. However, it should be noted that this study used a distinct set of tasks, so the three‐factor solution requires further validation. Moreover, the results highlight the trajectory of EFs development during the transition from kindergarten to school. The components of EFs continue to differentiate, as evidenced by the disappearance of correlations between visual and auditory‐verbal working memory, as well as between auditory‐verbal working memory and inhibitory control in second‐graders. Importantly, no significant differences were found between first‐ and second‐graders in any EFs components, suggesting a stabilization of EFs development during the initial years of schooling. This implies that the primary changes in EFs functioning occur during the transition from preschool to school age, while development becomes more gradual during the first 2 years of school (Romine and Reynolds 2005; Huizinga et al. 2006).

## 7 Limitations and Suggestions

Several limitations of this study should be noted. First, although a full assessment of the strength of correlations is only valid if the variables are measured on the same scale, it is worth mentioning that the correlations obtained in our study could be interpreted as weak yet significant. Such relationships were observed between cognitive flexibility, visual working memory, and inhibitory control across all age groups. Additionally, relatively weak correlation coefficients were found between auditory‐verbal working memory and inhibitory control in children up to the second grade. Second, different diagnostic tools were used to assess cognitive flexibility in senior preschool and elementary school children (in senior preschoolers (the DCCS) and in elementary school children (the NEPSY‐II “Inhibition” subtest, cognitive flexibility task)). Differences in methods may affect the comparability of the results obtained and limit interpretation. Third, in the latent factor structure of EFs for senior preschool children, the variance for the cognitive flexibility factor exceeded 100%. This conclusion is a well‐known statistical artifact in CFA. Similar effects have already been noted in studies of early childhood development (Huizinga et al. 2006). The probability of obtaining such results is further increased due to the high correlation between the indicators. Attempts to solve this problem by reducing the sample or limiting the model may lead to insufficient consistency and loss of theoretical confidence. We decided to keep the original approach to sampling and measurements. In future studies, it is planned to increase the sample size, which may help reduce the variance indicator (Fabrigar et al. 1999; MacCallum et al. 1999; Huizinga et al. 2006).

Regarding the prospects for studying the factor structure of EFs, it is important to investigate the existence of a fourth factor—planning—in Russian elementary school children. Additionally, it is crucial to trace the development of the factor structure of EFs in younger preschoolers, starting from the age of 3, and in third and fourth grade of elementary school children and beyond. Furthermore, conducting a longitudinal study of the factor structure of EFs would provide a deeper understanding of the trajectories and mechanisms underlying the development of all EFs components compared to cross‐sectional studies. Another limitation of this study is the lack of data on a number of family factors (parents' level of education, economic status, and family structure, etc.) that may influence the development of EFs in preschool and elementary school children. Measuring EFs using single tasks may be considered a research limitation. This is because it may increase the likelihood of a “task impurity effect,” when the success of the test depends on additional cognitive processes. This can potentially distort the structure of the factors. Therefore, we carefully interpret our results, particularly in terms of their generalizability, and we plan to overcome this limitation in our future longitudinal studies.

## 8 Conclusion

This study further clarified that a three‐factor model of EFs is the most parsimonious model for both senior preschool (senior and preparatory kindergarten groups) and elementary school children (first‐ and second‐graders). The findings support the three‐factor model of EFs proposed by Miyake and Friedman (2012), which describes the EFs construct as “unity and diversity.” The latent factor structure of EFs is characterized by predominantly moderate and robust correlations between EFs components within each age group. This study provided further clarification regarding the nature and composition of EFs in children. From a practical point of view, because the findings allow for a more differentiated approach to the diagnosis and correction of EFs. Thus, the presence of the three‐factor structure of EFs, starting at age 5, allows us to develop new diagnostic tools and targeted correctional programs, taking into account the specifics of the development of each component of EFs.

## Conflicts of Interest

The authors declare no conflicts of interest.

## Data Availability

Research data are not shared.
